# Impact of Annealing on Magnetic Properties and Structure of Co_40_Fe_40_W_20_ Thin Films on Si(100) Substrate

**DOI:** 10.3390/ma14113017

**Published:** 2021-06-02

**Authors:** Wen-Jen Liu, Yung-Huang Chang, Yuan-Tsung Chen, Tian-Yi Jhou, Ying-Hsuan Chen, Te-Ho Wu, Po-Wei Chi

**Affiliations:** 1 Department of Materials Science and Engineering, I-Shou University, Kaohsiung 840, Taiwan; jurgen@isu.edu.tw; 2 Bachelor Program in Interdisciplinary Studies, National Yunlin University of Science and Technology, 123 University Road, Section 3, Yunlin 64002, Taiwan; changyhu@yuntech.edu.tw; 3 Graduate School of Materials Science, National Yunlin University of Science and Technology, 123 University Road, Section 3, Yunlin 64002, Taiwan; M10847001@yuntech.edu.tw (T.-Y.J.); M10947013@yuntech.edu.tw (Y.-H.C.); wuth@yuntech.edu.tw (T.-H.W.); 4 Institute of Physics, Academia Sinica, Nankang, Taipei 11529, Taiwan; jacky01234567891@hotmail.com

**Keywords:** annealed Co_40_Fe_40_W_20_ thin films, low-frequency alternating current magnetic susceptibility, alternating-gradient magnetometer, X-ray diffraction, thermal stability

## Abstract

Co_40_Fe_40_W_20_ monolayers of different thicknesses were deposited on Si(100) substrates by DC magnetron sputtering, with Co_40_Fe_40_W_20_ thicknesses from 10 to 50 nm. Co_40_Fe_40_W_20_ thin films were annealed at three conditions (as-deposited, 250 °C, and 350 °C) for 1 h. The structural and magnetic properties were then examined by X-ray diffraction (XRD), low-frequency alternative-current magnetic susceptibility (χ_ac_), and an alternating-gradient magnetometer (AGM). The XRD results showed that the CoFe (110) peak was located at 2θ = 44.6°, but the metal oxide peaks appeared at 2θ = 38.3, 47.6, 54.5, and 56.3°, corresponding to Fe_2_O_3_ (320), WO_3_ (002), Co_2_O_3_ (422), and Co_2_O_3_ (511), respectively. The saturation magnetization (Ms) was calculated from the slope of the magnetization (M) versus the CoFeW thickness. The Ms values calculated in this manner were 648, 876, 874, and 801 emu/cm^3^ at the as-deposited condition and post-annealing conditions at 250, 350, and 400 °C, respectively. The maximum M_S_ was about 874 emu/cm^3^ at a thickness of 50 nm following annealing at 350 °C. It indicated that the M_S_ and the χ_ac_ values rose as the CoFeW thin films’ thickness increased. Owing to the thermal disturbance, the M_S_ and χ_ac_ values of CoFeW thin films after annealing at 350 °C were comparatively higher than at other annealing temperatures. More importantly, the Co_40_Fe_40_W_20_ films exhibited a good thermal stability. Therefore, replacing the magnetic layer with a CoFeW film improves thermal stability and is beneficial for electrode and strain gauge applications.

## 1. Introduction

Ever since the discovery of Co_50_Fe_50_ by Ellis in 1927 and Elmen in 1929, it has been shown that it has good soft magnetic properties [1]. The soft magnetic material of the Co–Fe system has been extensively applied in read heads of hard disks and magnetoresistive random access memories (MRAMs) because of their good mechanical properties, high spin polarization, high Curie temperature (T_c_), high saturation magnetization (M_s_), low coercivity (H_c_), and high spin polarization [2,3,4,5,6,7,8]. The CoFeB layer was utilized to combine with the MgO layer to form magnetic tunnel junctions (MTJs). For an MTJ system, being perpendicular magnetic anisotropic (PMA) is indispensable. However, one obstacle is the degradation of PMA properties at high temperatures. Recently, researchers have focused on increasing perpendicular magnetic anisotropic properties and thermal stability and improving the mechanical properties of MTJs’ structure. To insert other metal spacer layers into an MTJ system is common and effective at improving its thermal stability [9,10,11]. Another way to enhance PMA properties is to use multilayer structures, but this method causes the multilayer films’ whole thickness to increase. Transition metal (TM) alloys have a unique capability that can be used to improve some properties of magnetic films, including their thermal stability, mechanical strength, and chemical and physical properties [12,13,14,15,16,17,18]. Ghaferi et al. used a citrate bath to test a CoFeW alloy. Subsequent researchers found a variation of the W content with a pH value of different concentrations in 2016 [19]. Pai et al. studied the phase transition thickness of rare earth transition metal W. The results showed that the effect of the spin Hall angle is critical when W is employed as seed layer [20]. The addition of W in CoFe materials has some benefits such as durability, corrosion resistance, and thermal stability [21,22]. For magnetic research, CoFeW is a novel material. It is therefore attractive to research the characteristics of thin CoFeW films deposited by DC magnetron sputtering at as-deposited and annealed conditions. The various thicknesses (t_f_) of as-deposited and annealed CoFeW films and, therefore, the effect of crystallinity on the magnetic properties of CoFeW films were also studied. In our previous research, the as-deposited and post-annealing glass/CoFeW were examined for their magnetic and crystallinity properties, as mentioned in Table 1 [23]. In this study, it was found that the thermal stability of Co_40_Fe_40_W_20_ was about 350 °C. However, in order to apply a magnetic film in MRAM, the 400 °C of thermal tolerance is required for complementary metal-oxide-semiconductor (CMOS) and back-end-of-line (BEOL) process compatibility [22,24]. The thermal tolerance of Co_40_Fe_40_W_20_ is less than 400 °C. CoFeW film can be used as an electrode and is compatible with the whole device on the substrate after annealing at 300 °C [25]. Moreover, the Co_40_Fe_40_W_20_ film also offers the potential for high sensitivity for strain gauge application [26].

## 2. Materials and Methods

CoFeW with a thickness of 10–50 nm was sputtered onto an Si(100) substrate at room temperature (RT) by the magnetron DC sputtering direct method with 50 W power and under the subsequent four conditions: (a) the deposited films were kept at RT, (b) annealed at a treatment temperature (T_A_) at 250 °C for 1h, and (c) annealed at 350 °C for 1 h. The chamber base pressure was 2 × 10^−7^ Torr, and, therefore, the Ar working pressure was 3 × 10^−3^ Torr. The pressure in the ex-situ annealed condition was 3 × 10^−3^ Torr with a selected Ar gas. The alloy target of the CoFeW composition was 40 at% Co, 40 at% Fe, and 20 at% W. Co_40_Fe_40_B_20_ thin films are often used as free layers or pinned layers to make MTJ with an MgO layer [27,28]. The most common purpose of selecting the CoFeW composition is to explore the comparison of the roles of B and W. The structure of CoFeW thin films was detected by grazing incidence X-ray diffraction (GIXRD) patterns obtained with CuKα1 (PAN analytical X’pert PRO MRD) and a low-angle diffraction incidence (about a two-degree angle). The in-plane low-frequency alternate-current magnetic susceptibility (χ_ac_) and the hysteresis loop of Co_40_Fe_40_W_20_ were studied by an χ_ac_ analyzer (XacQuan) and an alternating-gradient magnetometer (AGM). To research the thermal tolerance, the saturation magnetization (M_s_) of the post-annealing 400 °C condition was calculated from the slope of the magnetization (M) versus the CoFeW thickness. Moreover, in χ_ac_ measurement, the χ_ac_ analyzer was used to calibrate the standard sample under the action of an external magnetic field. Then, the sample was inserted into the χ_ac_ analyzer. The driving frequency was between 10 and 25,000 Hz. χ_ac_ was measured by magnetization. All test samples had an equivalent shape and size to eliminate demagnetization. The χ_ac_ valve was an arbitrary unit (a.u.) because the AC result corresponded to the reference standard sample and may be a comparison value. The connection between magnetic susceptibility and frequency was measured by an χ_ac_ analyzer. The best resonance frequency (f_res_) was measured by an χ_ac_ analyzer and represents the frequency of the maximum χ_ac_.

## 3. Results

### 3.1. X-ray Diffraction

**Figure 1:**
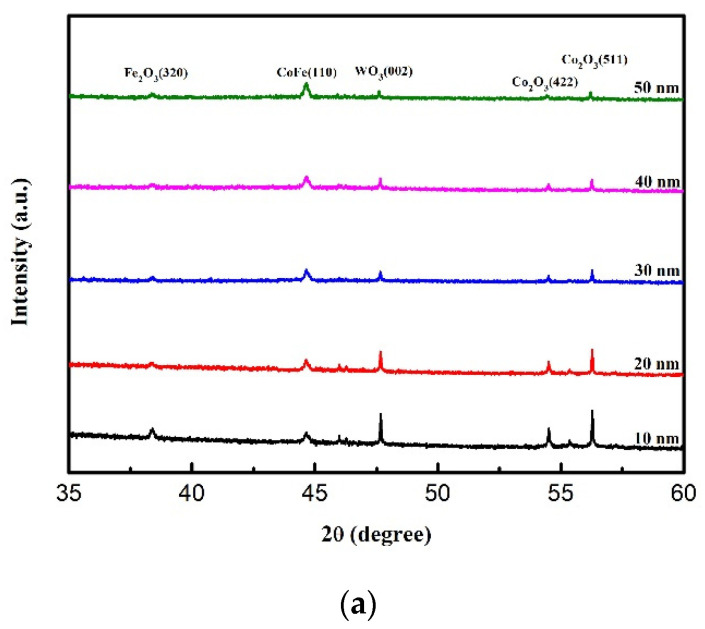

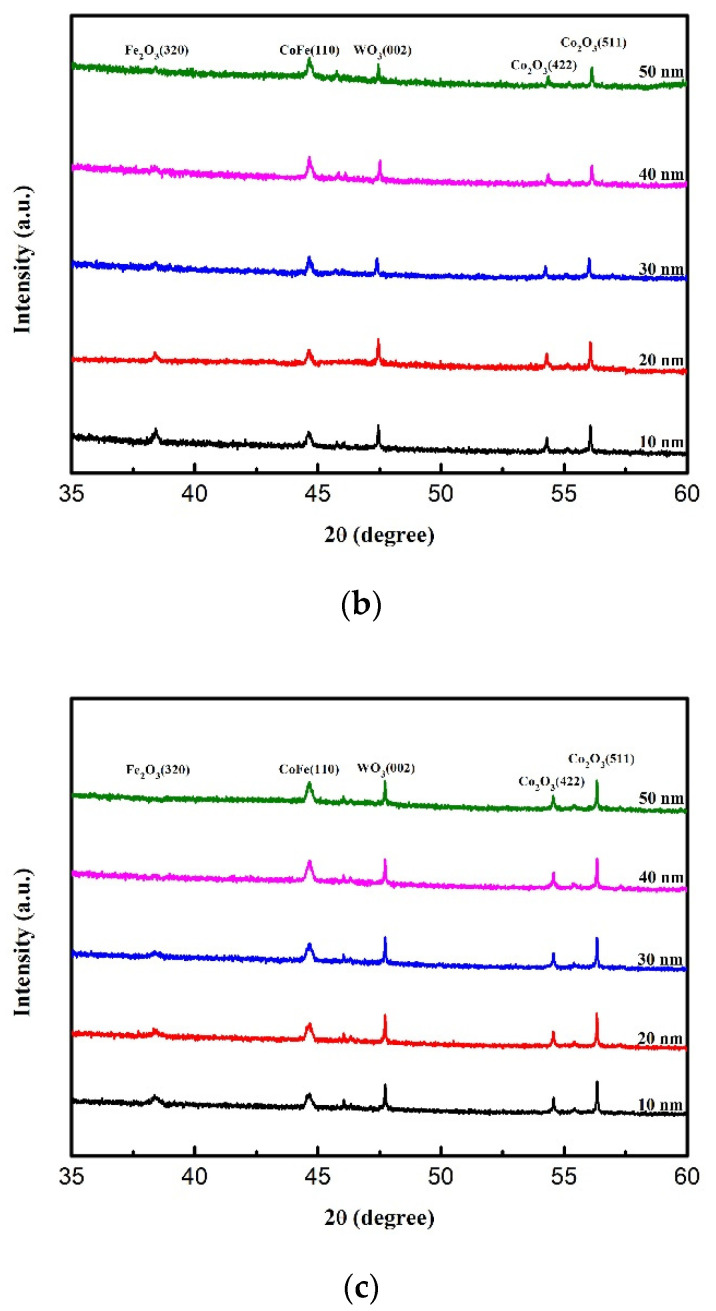
X-ray diffraction patterns of CoFeW thin films. (**a**) As-deposited, (**b**) post-annealing at 250 °C, (**c**) post-annealing at 350 °C.

Figure 1 displays the XRD patterns of as-deposited and annealed Si(100)/Co_40_Fe_40_W_20_ thin films with a thickness of 10 to 50 nm. Figure 1a shows the patterns of thin films that were formed as-deposited, whereas those that were formed post-annealing at 250 and 350 °C are displayed in Figure 1b,c. The XRD is presented at diffracted angles (2θ) between 35 and 60 degrees. The CoFe (110) peaks are exhibited, which could be clearly detected at around 2θ = 44°, indicating that the CoFeW thin films belonged to a crystallized state. It was generally observed in CoFeW thin films that the intensity of the CoFe(110) peaks increased with greater thickness. The specific oxide peaks appeared at 2θ = 38.3, 47.6, 54.5, and 56.3° in all CoFeW samples. They corresponded to Fe_2_O_3_ (320), WO_3_ (002), Co_2_O_3_ (422), and Co_2_O_3_ (511). Although the chamber was pumped to 10^−7^ Torr within the sputtering system, oxygen may still have been present. Both natural oxides on the Si(100) substrate and oxygen contamination on the sputtering targets contributed to the formation of oxidation peaks [29]. As CoFeW thicknesses increased, we found that the intensity of all oxide peaks decreased. It was speculated that the thickness of oxide was substantially the same in CoFeW thin films. Figure 1a–c demonstrates that the intensity of CoFe (110) peaks increased as the post-annealing temperature increased. According to [23], it was suggested that the crystallization of as-deposited CoFeW films on Si(100) substrate was better than ones on glass substrate.

### 3.2. Magnetic Analysis

**Figure 2:**
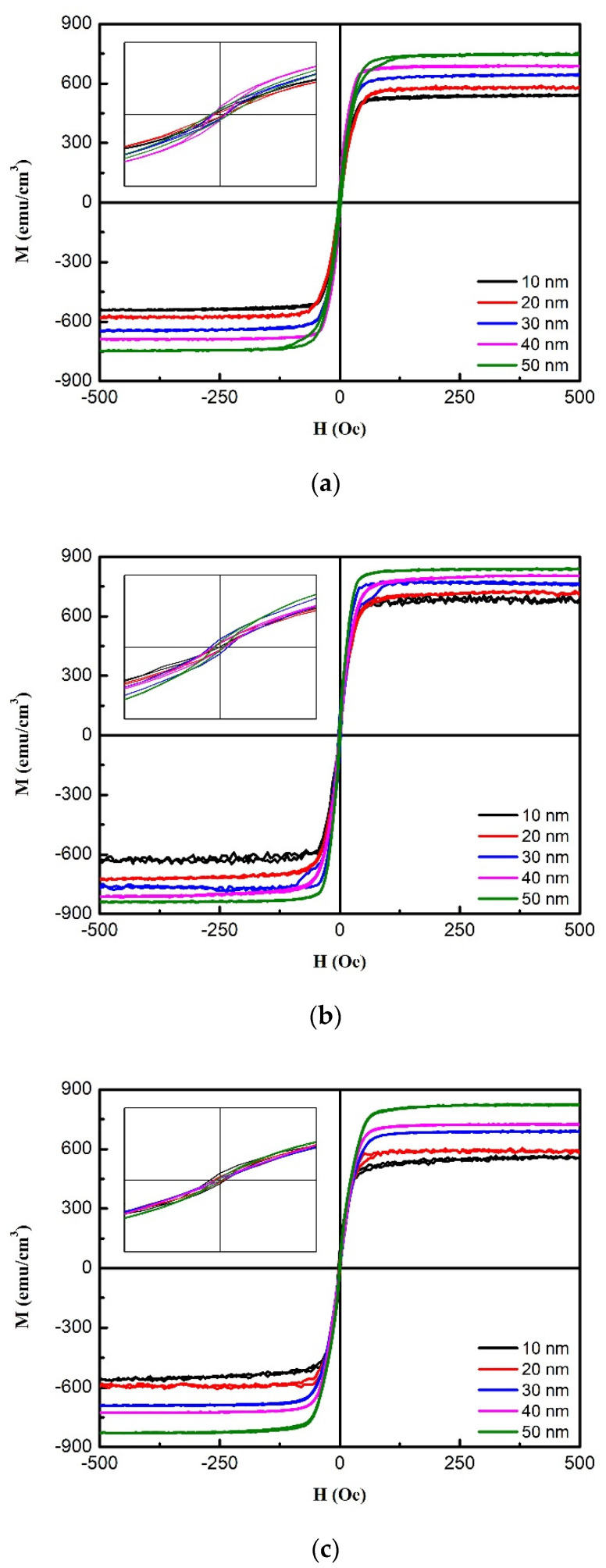
In-plane magnetic hysteresis loop of CoFeW thin films. (**a**) As-deposited, (**b**) post-annealing at 250 °C, (**c**) post-annealing at 350 °C.

Figure 2a–c displays the magnetic hysteresis loops of the CoFeW thin films under the three annealed conditions with thicknesses from 10 to 50 nm. The external magnetic field of 500 Oe in the plane was enough to observe the saturation magnetic spin state. The enlarged figure shows low coercivity (H_C_), which indicates that he CoFeW films were soft magnetic. The saturation magnetization (M_S_) of the CoFeW thin films under the three post-annealing conditions illustrates the magnetic properties of the CoFeW thin film, which were measured by the AGM, as shown in Figure 2. The M_S_ of the CoFeW thin films is summarized in Table 2. CoFeW films showed an in-plane magnetization in this study because the CoFeW film was too thick and was deposited on the Si substrate, which was due to the perpendicular magnetic anisotropy properties that originated from the Fe–O bond and the in-plane demagnetization field, which was too large owing to the thick CoFeW [30,31].

**Figure 3:**
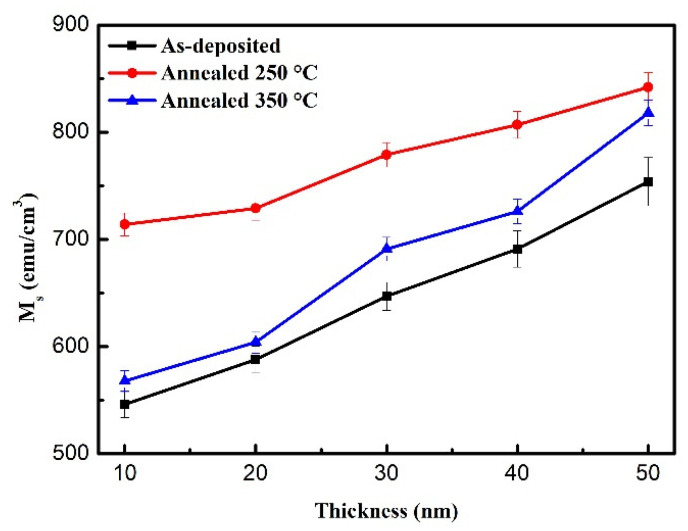
Saturation magnetization (M_S_) of CoFeW thin films.

Figure 3 shows the saturation magnetization (M_S_) of the CoFeW film with the as-deposited state and two post-annealing conditions. The results display that the M_S_ increased as a function of the thicknesses and indicate the thickness effect of M_S_ in CoFeW thin films.

**Figure 4:**
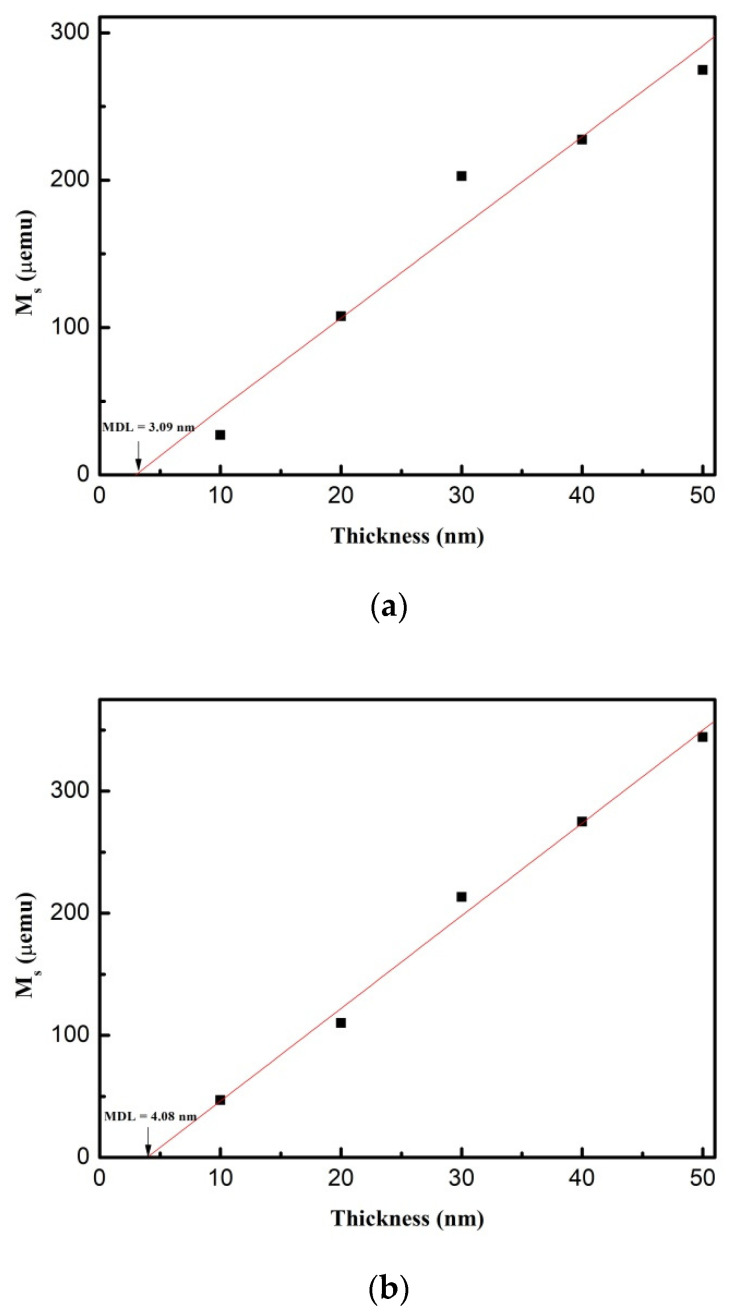

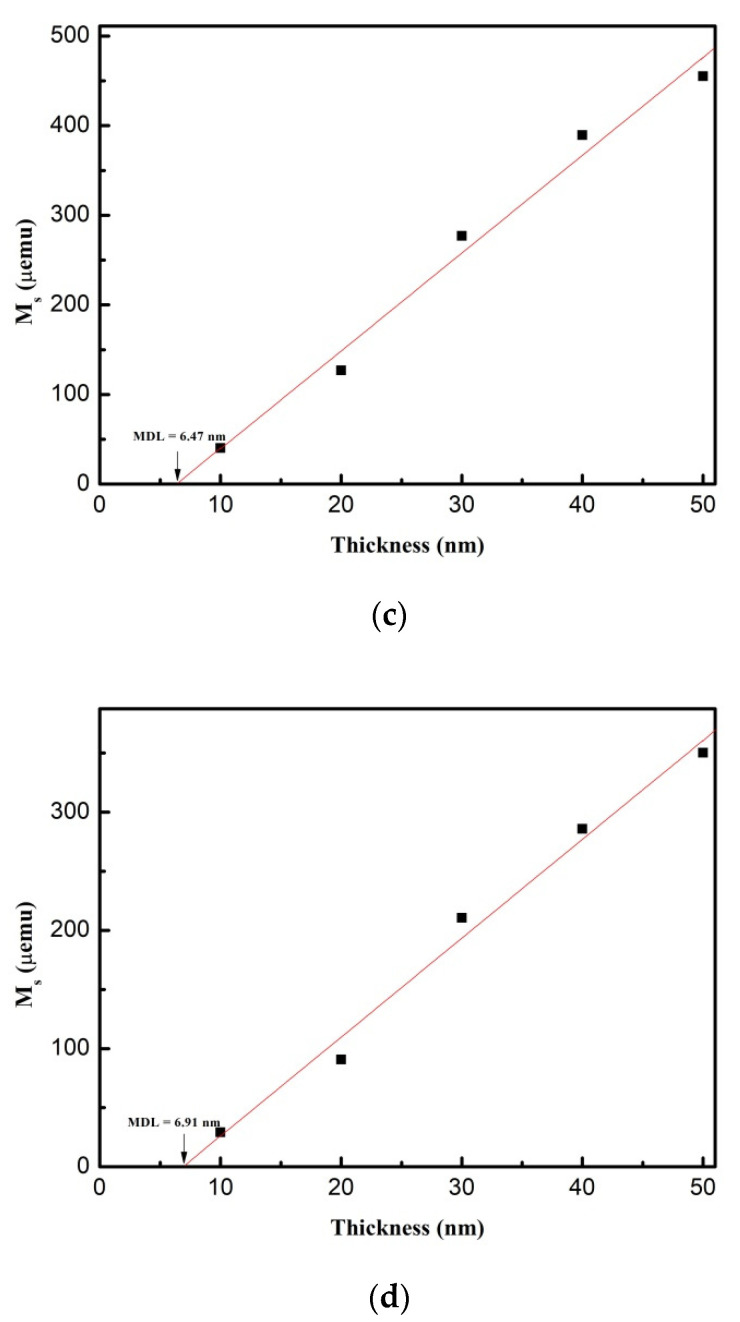
Magnetic dead layer thickness of CoFeW thin films. (**a**) As-deposited, (**b**) post-annealing at 250 °C, (**c**) post-annealing at 350 °C, (**d**) post-annealing at 400 °C.

The magnetic dead layer (MDL) thickness of as-deposited and post-annealing conditions was obtained from thickness fitting the intercept in the magnetization versus thickness plot, which is shown in Figure 4a–d. From the result, the MDL thickness of post-annealing CoFeW thin films was thicker than the as-deposited films owing to the annealing effect. The MDL thicknesses, obtained from Figure 4, were 3.09, 4.08, 6.47, and 6.91 nm at the as-deposited condition and the post-annealing conditions at 250, 350, and 400 °C, respectively. The natural oxidation layer of the Si surface was not removed before depositing the CoFeW film. However, the thickness of the native oxidation layer was 2 nm. The magnetic dead layer was detected in the interface. It can be reasonably concluded that there was a magnetic dead layer at the bottom Si(100) surface [32]. The influence of the natural oxidation layer on the magnetic properties was large enough because the magnetic dead layer thickness was comparable to the natural oxidation layer on the Si substrate as mentioned above. It can be reasonably concluded that antiferromagnetic oxidation was formed in the CoFeW film. To obtain the Ms of CoFeW, the MDL must be considered because the MDL thickness is not negligible compared to the CoFeW thickness. In this case, the Ms should be calculated from the slope of the M versus the CoFeW thickness. The Ms calculated from this manner was 648, 876, 874, and 801 emu/cm^3^ at the as-deposited condition and the post-annealing conditions at 250, 350, and 400 °C, respectively. These results indicate that the Ms value was almost the same at the annealed conditions at 250 and 350 °C. Therefore, the thermal tolerance of CoFeW was not 250 °C but more than or equal to 350 °C. Furthermore, it suggested that the M_S_ of the CoFeW thin films increased when raising the post-annealing temperature. There was an important correlation between all conditions of the M_S_, the temperature, and the thickness. These results indicate that the highest M_S_ after post-annealing was at 350 °C, which was the best heat-resistant temperature in this research. The oxidation of all CoFeW thin films on Si(100) substrate was better than those on glass substrate [24]. The results showed that the Ms of CoFeW films decreases obviously under various conditions. This means that the oxide was unfavorable for the M_S_ of the CoFeW thin films. According to the fitting result of Figure 4, the M_S_ value of Co_40_Fe_40_W_20_ thin films was increased to 350 °C, then decreased to 400 °C, which shows that the thermal stability of Co_40_Fe_40_W_20_ thin films is better than that found in other research [14]. The thermal tolerance of Co_40_Fe_40_W_20_ was less than 400 °C. The results show that Co_40_Fe_40_W_20_ film is suitable for electrode and strain gauge applications.

**Figure 5:**
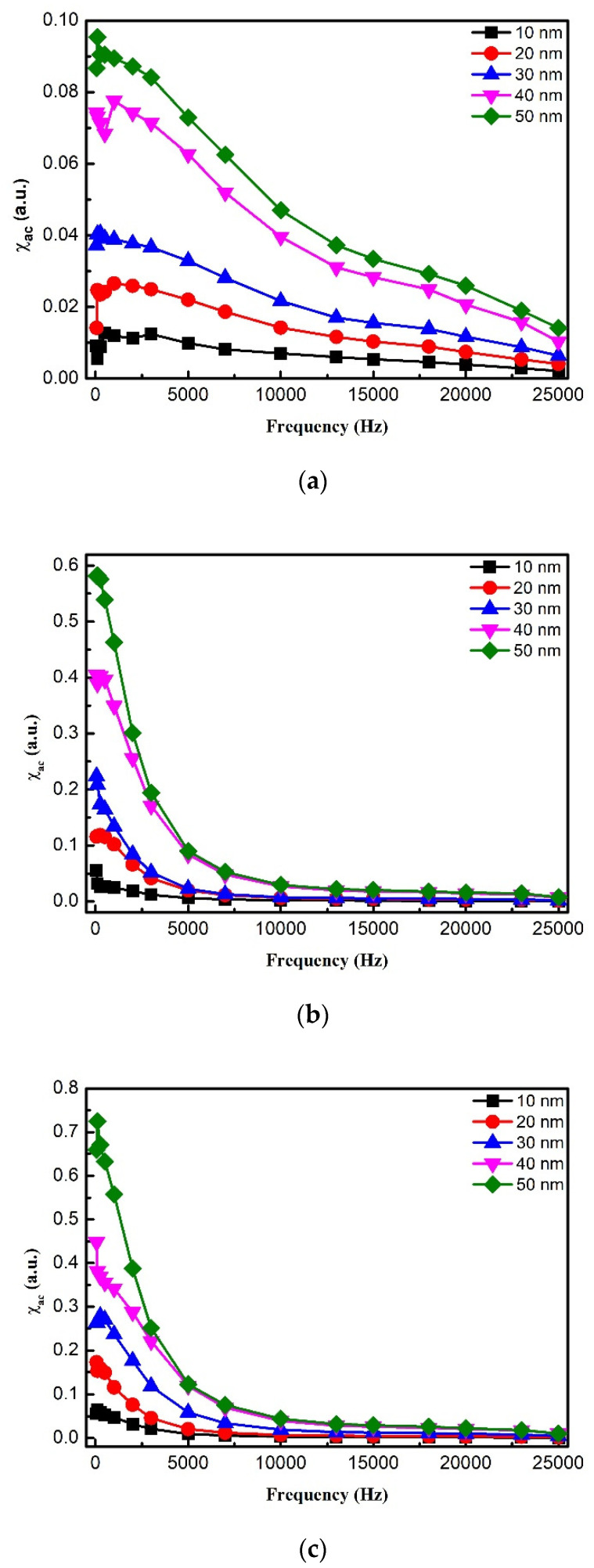
The low-frequency alternate-current magnetic susceptibility (χ_ac_) as a function of the frequency from 50 to 25,000 Hz. (**a**) As-deposited, (**b**) post-annealing at 250 °C, (**c**) post-annealing at 350 °C.

Figure 5a depicts the χ_ac_ of as-deposited CoFeW thin films as a function of the frequency from 50 to 25,000 Hz for 10, 20, 30, 40, and 50 nm. Figure 5b,c presents the χ_ac_ of the post-annealing CoFeW samples that were annealed at 250 and 350 °C for 1 h versus the frequency ranging from 50 to 25,000 Hz for each CoFeW thin film’s thickness. The low frequencies were in the range of 50 to 25,000 Hz. The thickness of CoFeW film ranged from 10 to 50 nm, and the χ_ac_ values of CoFeW thin films decreased with increasing frequency under three conditions.

**Figure 6:**
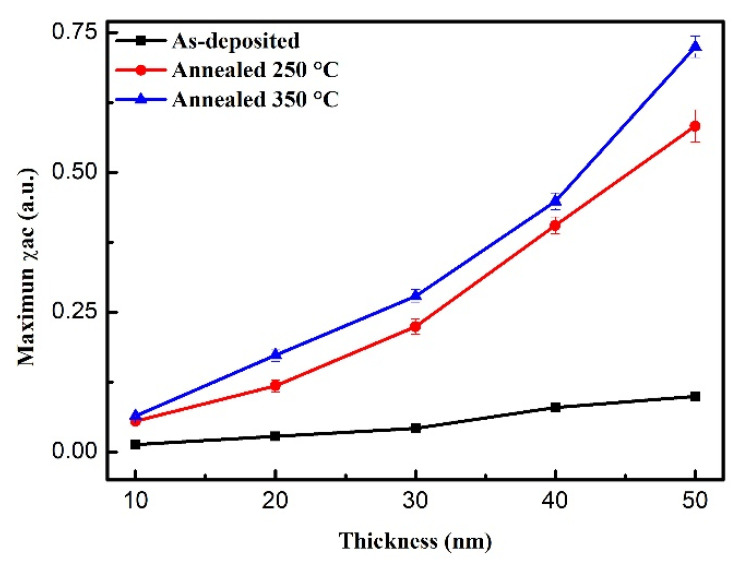
Maximum alternate-current magnetic susceptibility for the CoFeW thin films.

The corresponding maximum χ_ac_ values of different CoFeW thicknesses under three conditions are shown in Figure 6. The results reveal that the χ_ac_ values of the CoFeW films decreased with the increase in thickness. The χ_ac_ values of all CoFeW films dropped sharply at high frequency, as shown in Figure 6. The as-deposited CoFeW thin films showed that the maximum χ_ac_ value was 0.095 when the thickness was 50 nm. The post-annealing condition at 250 °C for the CoFeW thin films showed that the maximum χ_ac_ value was 0.583 when the thickness was 50 nm. The post-annealing condition of 350 °C for the CoFeW thin films showed that the maximum χ_ac_ value was 0.725 when the thickness was 50 nm. The results clearly reveal the thickness effect of χ_ac_ in all CoFeW samples. As the thickness of the CoFeW films increased, the maximum χ_ac_ value was augmented because of the thickness effect. The maximum χ_ac_ value of the CoFeW thin films after annealing was larger than the as-deposited samples. When the magneto-crystalline anisotropy of the CoFe (110) crystallization effect was maximized, the χ_ac_ value of CoFeW was maximized [33,34].

Table 3 represents the optimal resonance frequency (ƒ_res_) of the CoFeW thin films. When the maximum χ_ac_ value was presented with the ƒ_res_ value, the spin sensitivity was exceptional. Furthermore, when the ƒ_res_ value was below 1000 Hz for the thin films, it created CoFeW magnetic thin films for applications in the field of soft magnetism devices. Table 3 presents that the ƒ_res_ values of all CoFeW thicknesses, which were from 50 Hz to 1000 Hz. This means that the maximum χ_ac_ had the strongest spin sensitivity at this frequency [35,36].

## 4. Conclusions

The XRD patterns revealed that the CoFeW thin films are composed of a nanocrystalline body-centered cubic (BCC) CoFe phase and that they contain several Fe-, W-, and Co-based oxides. The intensity of CoFe (110) peaks generally increased with increasing film thicknesses, indicating a development of the crystallographic texture. Moreover, increasing the film thickness was accompanied by a reduced intensity in the diffraction peaks originating from oxides. An oxidation caused an increase in film thickness after annealing. The CoFeW films exhibited soft magnetism owing to the low H_c_ and in-plane magnetization; thicker CoFeW thicknesses showed a large in-plane demagnetization field and a weak Fe–O bonding effect. The MDL thickness of the post-annealing CoFeW thin films was thicker than the as-deposited films because of oxidation and the annealing effect. The Ms should be fitted from the slope of the M versus the CoFeW thickness and obtained 648, 876, 874, and 801 emu/cm^3^ at the as-deposited condition and the post-annealing conditions at 250, 350, and 400 °C, respectively. Alloying additions of W improved the thermal stability of the CoFe films. The maximum Ms and the maximum χ_ac_ value of 0.725 were achieved for CoFeW films with a thickness of 50 nm after annealing at 350 °C. The f_res_ values of all films being less than 1000 Hz confirmed that the CoFeW magnetic films are suitable for magnetic component applications.

## Figures and Tables

**Table 1:** Significant properties for glass/CoFeW and Si(100)/CoFeW materials.

Material	Thickness	Maximum χ_ac_ (a.u.)	Optimal Resonance Frequency, f_res_ (Hz)	Crystallinity
Glass/Co_32_Fe_30_W_38_ [23]	10–50 nm at RT and annealed conditions	0.02–0.52	50–1000	Weak
Si(100)/Co_40_Fe_40_W_20_	10–50 nm at RT and annealed conditions	0.055–0.745	50–1000	Strong

**Table 2:** Saturation magnetization (M_S_) values of CoFeW films from in-plane magnetic hysteresis loop.

Thickness (nm)	As-Deposited (emu/cm^3^)	Post-Annealing at 250 °C (emu/cm^3^)	Post-Annealing at 350 °C (emu/cm^3^)
10	546	714	568
20	588	729	604
30	647	779	691
40	691	807	726
50	754	842	818

**Table 3:** Optimal resonance frequency for films of various thicknesses.

Thickness (nm)	As-Deposited Optimal Resonance Frequency (Hz)	Post-Annealing at 250 °C of Optimal Resonance Frequency (Hz)	Post-Annealing at 350 °C of Optimal Resonance Frequency (Hz)
10	500	50	100
20	1000	250	50
30	250	50	250
40	1000	50	50
50	100	100	100

## Data Availability

The data presented in this study are available on reasonable request from the corresponding author.

## References

[1] Elmen G.W. (1929). Magnetic alloys of iron, nickel, and cobalt. J. Frankl. Inst..

[2] Li M., Wang S., Zhang S., Fang S., Yu G. (2019). The perpendicular magnetic anisotropies of CoFeB/MgO films with Nb buffer layers. J. Magn. Magn. Mater..

[3] Manos O., Bougiatioti P., Dyck D., Huebner T., Rott K., Schmalhorst J.M., Reiss G. (2019). Correlation of tunnel magnetoresistance with the magnetic properties in perpendicular CoFeB-based junctions with exchange bias. J. Appl. Phys..

[4] Sun J.Z., Trouilloud P.L., Lauer G.P., Hashemi P. (2019). Bias dependent conductance in CoFeB-MgO-CoFeB magnetic tunnel junctions as an indicator for electrode magnetic condition at barrier interfaces. AIP Adv..

[5] Ota1 S., Ono M., Matsumoto H., Ando A., Sekitani T., Kohno R., Iguchi S., Koyama T., Chiba D. (2019). CoFeB/MgO-based magnetic tunnel junction directly formed on a flexible substrate. Appl. Phys. Express.

[6] Huang S.X., Chen T.Y., Chien C.L. (2008). Spin polarization of amorphous CoFeB determined by point-contact Andreev reflection. Appl. Phys. Lett..

[7] Kalu E.E., Bell R., Dupree M. (2010). Improvement of the corrosion behavior of electrodeposited CoFeCu thin films. Mater. Chem. Phys..

[8] Kumari T.P., Raja M.M., Kumar A., Srinath S., Kamat S.V. (2014). Effect of thickness on structure, microstructure residual stress and soft magnetic properties of DC sputtered Fe_65_Co_35_ soft magnetic thin films. J. Magn Magn. Mater..

[9] Gottwald M., Lee K., Kan J.J., Ocker B., Wrona J., Tibus S., Langer J., Kang S.H., Fullerton E.E. (2013). Ultra-thin Co-Pd multilayers with enhanced high-temperature annealing stability. Appl. Phys. Lett..

[10] An G.G., Lee J.B., Yang S.M., Kim J.H., Chung W.S., Yoon K.S., Hong J.P. (2015). Correlation between Pd metal thickness and thermally stable perpendicular magnetic anisotropy features in [Co/Pd]n multilayers at annealing temperatures up to 500 °C. AIP Adv..

[11] Kawahara T., Ito K., Takemura R., Ohno H. (2012). Spin-transfer torque RAM technology: Review and prospect. Microelectron. Reliab..

[12] Wang S., Li M., Zhang S., Fang S., Wang D., Yu G. (2019). High annealing tolerance and the microstructure study in perpendicular magnetized MgO/CoFeB/MgO structures with thin W spacer layer. J. Magn. Magn. Mater..

[13] Liu Y., Yu T., Zhu Z., Zhong H., Khamis K.M., Zhu K. (2016). High thermal stability in W/MgO/CoFeB/W/CoFeB/W stacks via ultrathin W insertion with perpendicular magnetic anisotropy. J. Magn. Magn. Mater..

[14] Miyakawa N., Worledge D.C., Kita K. (2013). Impact of Ta Diffusion on the Perpendicular Magnetic Anisotropy of Ta/CoFeB/MgO. IEEE Magn. Lett..

[15] An G.G., Lee J.B., Yang S.M., Kim J.H., Chung W.S., Hong J.P. (2015). Highly stable perpendicular magnetic anisotropies of CoFeB/MgO frames employing W buffer and capping layers. Acta Mater..

[16] Sun J., Li H., Huang Y., Zhuang Z. (2018). CoFeW ternary oxides nanoparticles for oxygen evolution reaction. Mater. Lett..

[17] Meng H., Lum W.H., Sbiaa R., Lua S.Y.H., Tan H.K. (2011). Annealing effects on CoFeB-MgO magnetic tunnel junctions with perpendicular anisotropy. J. Appl. Phys..

[18] Kaidatzis A., Bran C., Psycharis V., Vázquez M., Martín J.M.G., Niarchos D. (2015). Tailoring the magnetic anisotropy of CoFeB/MgO stacks onto W with a Ta buffer layer. Appl. Phys. Lett..

[19] Ghaferi Z., Sharafi S., Bahrololoom M.E. (2016). The role of electrolyte pH on phase evolution and magnetic properties of CoFeW codeposited films. Appl. Surf. Sci..

[20] Pai C.F., Liu L., Li Y., Tseng H.W., Ralph D.C., Buhrman R.A. (2012). Spin transfer torque devices utilizing the giant spin Hall effect of tungsten. Appl. Phys. Lett..

[21] Almasi H., Sun C.L., Li X., Newhouse-Illige T., Bi C., Price K.C., Nahar S., Grezes C., Hu Q., Amiri P.K. (2017). Perpendicular magnetic tunnel junction with W seed and capping layers. J. Appl. Phys..

[22] Kim J.H., Lee J.B., An G.G., Yang S.M., Chung W.S., Park H.S., Hong J.P. (2015). Ultrathin W space layer-enabled thermal stability enhancement in a perpendicular MgO/CoFeB/W/CoFeB/MgO recording frame. Sci. Rep..

[23] Liu W.J., Chang Y.H., Ou S.L., Chen Y.T., Li W.H., Jhou T.Y., Chu C.L., Wu T.H., Tseng S.W. (2020). Effect of annealing on the structural, magnetic, surface energy and optical properties of Co_32_Fe_30_W_38_ films deposited by direct-current magnetron sputtering. Coatings.

[24] Kim D.H., Park K.W., Park B.G. (2017). Enhanced tunnel magnetoresistance and electric field effect in CoFeB/MgO/CoFeB perpendicular tunnel junctions with W under layer. Curr. Appl. Phys..

[25] Wang H., Kou X., Wang S., Zhou J., Zhang X., Li J. (2011). Structures, magnetic properties and thermal stability of CoFeB/MgO films. Phys. Procedia.

[26] Wang D., Nordman C., Qian Z., Daughton J.M., Myers J. (2005). Magnetostriction effect of amorphous CoFeB thin films and application in spin-dependent tunnel junctions. J. Appl. Phys..

[27] Wang W.X., Yang Y., Naganuma H., Ando Y., Yu R.C., Han X.F. (2011). The perpendicular anisotropy of Co_40_Fe_40_B_20_ sandwiched between Ta and MgO layers and its application in CoFeB/MgO/CoFeB tunnel junction. Appl. Phys. Lett..

[28] He C., Navabi A., Shao Q., Yu G., Wu D., Zhu W., Zheng C., Li X., He Q.L., Razavi1 S.A. (2016). Spin-torque ferromagnetic resonance measurements utilizing spin Hall magnetoresistance in W/Co_40_Fe_40_B_20_/MgO structures. Appl. Phys. Lett..

[29] Liu W.J., Chang Y.H., Chen Y.T., Chiang Y.C., Liu Y.C., Wu T.H., Chi P.W. (2021). Effect of annealing on the structural, magnetic, surface energy of CoFeBY films on Si (100) substrate. Materials.

[30] Iihama S., Mizukami S., Naganuma H., Oogane M., Ando Y., Miyazaki T. (2014). Gilbert damping constants of Ta/CoFeB/MgO(Ta) thin films measured by optical detection of precessional magnetization dynamics. Phys. Rev. B.

[31] Li M., Wang S., Zhang S., Fang S., Feng G., Cao X., Zhang P., Wang B., Yu G. (2019). The effect of interfacial oxygen migration on the PMA and thermal stability in MTJ with double MgO layers. Appl. Surf. Sci..

[32] Jang S.Y., Lim S.H., Lee S.R. (2010). Magnetic dead layer in amorphous CoFeB layers with various top and bottom structures. J. Appl. Phys..

[33] Budde T., Gatzen H.H. (2004). Magnetic properties of an SmCo/NiFe system for magnetic microactuators. J. Magn. Magn. Mater..

[34] Wen D., Li J., Gan G., Yang Y., Zhang H., Liu Y. (2019). Double peaks of the permeability spectra of obliquely sputtered CoFeB amorphous films. Mater. Res. Bull..

[35] Yang S.Y., Chien J.J., Wang W.C., Yu C.Y., Hing N.S., Hong H.E., Hong C.Y., Yang H.C., Chang C.F., Lin H.Y. (2011). Magnetic nanoparticles for high-sensitivity detection on nucleic acids via superconducting-quantum-interference-device-based immunomagnetic reduction assay. J. Magn. Magn. Mater..

[36] Chen Y.T., Xie S.M., Jheng H.Y. (2013). The low-frequency alternative-current magnetic susceptibility and electrical properties of Si(100)/Fe_40_Pd_40_B_20_(X Å)/ZnO(500 Å) and Si(100)/ZnO(500 Å)/Fe_40_Pd_40_B_20_(Y Å) systems. J. Appl. Phys..

